# A report of dangerously high carbon monoxide levels within the passenger compartment of a snow-obstructed vehicle

**DOI:** 10.1186/1471-227X-4-4

**Published:** 2004-10-10

**Authors:** Jill A Griffin, Stephen J Playe, Benjamin Osborne, Howard A Smithline

**Affiliations:** 1 Department of Emergency Medicine, Tufts University School of Medicine, Baystate Medical Center, 759 Chestnut Street, Springfield, MA, 01199 USA

## Abstract

**Background:**

We sought to determine how quickly carbon monoxide would accumulate in the passenger compartment of a snow-obstructed vehicle.

**Methods:**

A 1992 sedan was buried in snow to the level of the undercarriage, the ignition was then engaged and carbon monoxide levels recorded at 2.5-minute intervals. The primary outcome was the time at which a lethal carbon monoxide level was detected. Six trials were conducted: windows closed; windows open one inch; windows open 6 inches; windows closed and tailpipe swept clear of snow; windows closed and one cubic foot of snow removed around tailpipe; windows closed and tailpipe completely cleared of snow to ground level in a path 12 inches wide.

**Results:**

Lethal levels of carbon monoxide occurred within 2.5 minutes in the vehicle when the windows were closed, within 5 minutes when the widows were opened one inch, and within 7.5 minutes when the widows were opened six inches. Dangerously high levels of carbon monoxide were detected within the vehicle when the tailpipe had been swept clear of snow and when a one cubic foot area had been cleared around the tailpipe. When the tailpipe was completely unobstructed the carbon monoxide level was zero.

**Conclusions:**

Lethal levels of carbon monoxide occurred within minutes in this snow-obstructed vehicle.

## Background

Carbon monoxide poisoning is the number one cause of toxin related death in the United States [1]. It has been estimated that this poison may be responsible for up to 1,500 accidental deaths and 10,000 medical visits annually in the United States [2].

One of the manners in which people die from carbon monoxide poisoning is hypoxia secondary to entrapment within a snow-obstructed vehicle. Recommendations for prevention of these poisonings has been well documented but not thoroughly studied. The Centers for Disease Control (CDC) recommends that "following heavy snowfall, the public should be reminded to inspect vehicles to ensure that exhaust pipes are cleared of snow before engines are started" [3]. Brian Horner, in the Wilderness Medicine Letter made the following recommendation "The windows should be rolled down one inch on each side to provide cross-ventilation. Check the exhaust tail-pipe frequently to see that it is free of drifting snow" [4]. Both the Federal Emergency Management Agency (FEMA) [5] and the National Oceanic and Atmospheric Administration (NOAA) [6] recommend keeping the tailpipe clear and partially opening a window to prevent carbon monoxide poisoning.

In reviewing case reports regarding snow emergencies, it appears that there have been situations where venting carbon monoxide through an open window may not have been sufficient to prevent dangerously high levels of carbon monoxide from accumulating within the passenger compartment [7]. This raises the question of whether current prevention guidelines are safe and accurate.

We performed a pilot study with a single vehicle to simulate a snow emergency whereby a person stranded in a vehicle during a snowstorm would perform a safety maneuver. Our hypothesis was that opening a window a few inches for ventilation or clearing a tailpipe of snow would be sufficient to keep carbon monoxide levels in a non-lethal range.

## Methods

In 1995, a 1992 four-door sedan whose exhaust system had been inspected by the State of Massachusetts and found to be functioning normally was placed in a driveway and snow was shoveled around its base on all four sides until the car was obstructed by snow to the level of the bumpers. A Nighthawk, battery powered, digital readout, 80 mAmp carbon monoxide detector was attached to the front of the driver's seat headrest. The instrument had the ability to detect carbon monoxide levels in the range of zero to nine hundred ninety nine parts per million (0 – 999 ppm) [8]. The ignition was engaged and carbon monoxide levels were measured every two and a half minutes until either the maximum level of 999 ppm was detected or 10 minutes had passed.

Six trials were performed. Between each trial all doors and windows were opened, the carbon monoxide was allowed to exhaust from the vehicle until the detector registered zero parts per million.

## Results

Dangerously high carbon monoxide concentrations were recorded in the passenger compartment within three minutes when the windows were closed, within five minutes when the front windows were open one inch and within 7.5 minutes when the windows were opened six inches (Table 1). With the windows closed and tailpipe swept clear, a carbon monoxide level of 751 ppm was recorded at 7.5 minutes. With the windows closed and the tail pipe cleared one cubic foot around, the highest CO level recorded was 299 ppm at 10 minutes. When the tailpipe was completely cleared (12 inches wide to ground level) no carbon monoxide was detected.

**Table 1:** Carbon monoxide levels in a snow-obstructed vehicle.

Time (minutes)	Trial #1	Trial #2	Trial #3	Trial #4	Trial #5	Trial #6
0	0*	0	0	0	0	0
2.5	999	530	8	28	0	0
5		999	113	555	33	0
7.5			999	751	265	0
10				585	299	0
12.5				622	276	0

*Parts Per Million

Trial #1: Windows Closed; Trial #2: Windows Open one inch; Trial #3: Windows Open 6 inches; Trial #4: Windows Closed, Tailpipe brushed clear of snow; Trial #5: Windows Closed, Tailpipe brushed clear of snow in an area one cubic foot around tail-pipe; Trial #6: Windows Closed, Tailpipe brushed clear of snow in an area 12 inches wide, depth to ground level.

## Discussion and conclusions

Carbon monoxide is an odorless, tasteless by-product of fossil fuel combustion [1]. When it accumulates undetected in the passenger compartment of a snow obstructed vehicle it can rapidly cause toxicity and death. Toxic exposures increase during winter months in the United States and heavy snowfalls that occur over a short periods of time represent a potentially hazardous situation for travelers and for those who must remove snow from vehicles that have been parked outside [9].

In this study, only opening a window was not enough to prevent accumulation of carbon monoxide within the passenger compartment of the vehicle. As the guidelines suggest [3-6], snow must also be cleared from around the tailpipe. However, in this study, the passenger compartment was not safe until the tailpipe had been brushed clear of snow in an area twelve inches wide down to ground level. The major limitations of this study are the small sample size, the fact that the snow used in the trial was shoveled (higher density) rather than naturally accumulating, and that trial #1 was performed on a cold engine. Since multiple vehicles were not tested and since the temperature of the engine, air, and snow was not determined, it would be difficult to generalize the findings of this single vehicle study. Cold engines emit significantly higher levels of carbon monoxide at startup [10], all vehicles emit different levels of carbon monoxide at startup, and snow density and environmental conditions may effect carbon monoxide accumulation. Further studies to validate the study findings using in multiple vehicles at multiple operating temperatures are recommended.

## Competing interests

The authors declare that they have no competing interests.

## Authors' contributions

JG gathered data and prepared the manuscript. SP conceived of the project and gathered data. BO gathered data and presented the abstract at the Society for Academic Emergency Medicine. HS oversaw the project and provided statistical support.

## Pre-publication history

The pre-publication history for this paper can be accessed here:


